# The effects of low-impact mutations in digital organisms

**DOI:** 10.1186/1742-4682-8-9

**Published:** 2011-04-18

**Authors:** Chase W Nelson, John C Sanford

**Affiliations:** 1 Rainbow Technologies, Inc., 877 Marshall Rd., Waterloo, NY 13165, USA; 2 Department of Horticulture, NYSAES, Cornell University, Geneva, NY 14456, USA

## Abstract

**Background:**

Avida is a computer program that performs evolution experiments with digital organisms. Previous work has used the program to study the evolutionary origin of complex features, namely logic operations, but has consistently used extremely large mutational fitness effects. The present study uses Avida to better understand the role of low-impact mutations in evolution.

**Results:**

When mutational fitness effects were approximately 0.075 or less, no new logic operations evolved, and those that had previously evolved were lost. When fitness effects were approximately 0.2, only half of the operations evolved, reflecting a threshold for selection breakdown. In contrast, when Avida's default fitness effects were used, all operations routinely evolved to high frequencies and fitness increased by an average of 20 million in only 10,000 generations.

**Conclusions:**

Avidian organisms evolve new logic operations only when mutations producing them are assigned high-impact fitness effects. Furthermore, purifying selection cannot protect operations with low-impact benefits from mutational deterioration. These results suggest that selection breaks down for low-impact mutations below a certain fitness effect, the *selection threshold*. Experiments using biologically relevant parameter settings show the tendency for increasing genetic load to lead to loss of biological functionality. An understanding of such genetic deterioration is relevant to human disease, and may be applicable to the control of pathogens by use of lethal mutagenesis.

## Background

The standard explanation for the origin of biological complexity is that it arises through the Darwinian process of mutation and natural selection. Beneficial mutations accumulate through positive selection, and deleterious mutations tend to be eliminated by purifying selection. However, developments in genomics suggest theoretical problems with this view, and many features of living systems cannot be explained without recourse to nonadaptive processes [1-4].

Because of the slow pace of evolutionary change, it has generally been difficult to empirically test long-term evolutionary scenarios. A computational approach known as digital genetics [5,6] attempts to overcome this limitation by using *digital organisms*, short computer programs that replicate and compete in a virtual environment. Generations take only a few seconds, making it possible to observe the outcome of large numbers of mutation and replication events in relatively short periods of real time. Further, the user is able to alter parameters of interest (e.g., mutation rates) to observe their influence on important population factors (e.g., fitness).

Early versions of digital life culminated in the program Tierra [5], which demonstrated adaptive genome shrinkage, cooperation, and parasitism. Genomes were simulated as computer code, distinguishing the software from numerical simulation. Mutating digital organisms competed for computer processing time, undergoing adaptive change over many generations. Recognizing the importance of local interactions, the program Avida [7,8] advanced the field by implementing a virtual world in which organisms were housed on a two-dimensional grid and underwent interactions with neighbors.

Researchers have claimed a high degree of biological relevance for Avida, comparing its digital organisms to organic viruses [9]. Titles like "The biology of digital organisms" [10], "Evolution of biological complexity" [11], and "Testing Darwin" [12] evidence Avida's impact on biological theory. In addition to the evolution of biological complexity [11,13], the software has been used to study the evolution of sex [9,14,15], the evolution of altruism [16], the dynamics of long-term adaptation [17-21], ecosystem dynamics [19,22-24], and the effects of mutation on genetic architecture [14,25-28], among other topics.

Avida is used in the present study to better understand the evolutionary consequences of low-impact mutations in digital organisms. Though many studies report the occurrence of neutral mutations, Eyre-Walker & Keightley [29] note that:

... it seems unlikely that any mutation is truly neutral in the sense that it has no effect on fitness. All mutations must have some effect, even if that effect is vanishingly small. However, there is a class of mutations that we can term effectively neutral... As such, the definition of neutrality is operational rather than functional; it depends on whether natural selection is effective on the mutation in the population or the genomic context in which it segregates, not solely on the effect of the mutation on fitness.

This point applies to viruses as well as more complex systems [30]. The term *selection threshold *has been introduced [Gibson P, *et al.*, in preparation] to describe the mutational fitness effect that marks the "tipping point" between natural selection and random genetic drift in an evolving system. Mutations with fitness effects below this critical value are primarily affected by random genetic drift. One of the first to allude to this phenomenon was Muller [31], who noted: "There comes a level of advantage... that is too small to be effectively seized upon by selection."

The selection threshold is elevated by any factor that influences replication rate in a manner independent of the genotype, decreasing the efficacy of selection as more mutations behave in a neutral fashion. Population size has typically been the primary focus of these factors [32], and its role is described in Kimura's [1] well-known expression, |*s*| < 1/(2*N_e_*). This inequality states that random genetic drift will dominate a mutation's fate if its selection coefficient (*s*) is less than the reciprocal of twice the effective population size (*N_e_*). However, numerous other factors also influence the selection threshold, including environmental noise and developmental canalization, and the efficacy of selection is highly dependent on the complexity of the system under study.

The present study takes an empirical approach to determining the selection threshold by measuring the mutational fitness effect at which selection successfully captures half of the beneficial mutations that arise. Previous experiments using Avida have studied the evolutionary emergence of complex features resulting from high-impact beneficial mutations [13]. Avida's default settings provide mutational fitness effects of 1.0 - 31.0 for beneficial mutations that give rise to certain computational operations, where fitness effects are measured as *w *- 1, and *w *is the relative fitness of the organism expressing a given operation. For example, a mutation producing the NAND operation will multiply an organism's fitness by 2, corresponding to a fitness effect of 1.0. However, fitness effects this large are extremely rare in nature (see Discussion). In the present study, we approximate the selection threshold in Avida by performing experiments with more biologically common mutational fitness effects of 1.0 and below. The effects of low-impact mutations are explored and the biological relevance of digital life is discussed.

### Avida

An experiment with Avida begins by seeding a two-dimensional grid with a short computer program (the *ancestral organism*) that has been designed to self-replicate. By default, a 60 × 60 grid is seeded with a single Avidian organism that consists of 100 computational instructions. This artificial geography allows the population to grow to a maximum of 3,600 organisms. Avidians replicate asexually for approximately 10,000 generations, incurring an average of 0.85 mutations per genome per generation. Mutations randomly substitute, insert, or delete single instructions in an Avidian genome, drawing upon 26 available instructions defined in the software. The ancestral genome devotes about 15 instructions to the essential replication code, while the remaining 85 positions are occupied by benign *no-operation *instructions, analogous to inert "junk DNA" that can be used as raw material for evolutionary tinkering.

Once an experiment begins, replication ensues, and multiple organisms arise and compete with one another. When an Avidian replicates, its offspring is randomly placed in one of eight positions surrounding the parent organism, effectively killing the previous resident. Speed of replication therefore defines fitness in Avida; the programs that replicate fastest replace their slower counterparts and increase in number.

Speed of replication is itself determined by two factors. The first and primary way that Avidians replicate faster is by earning additional computer resources. The allocation of computer time is based upon an organism's *merit*, a numerical value that reflects its ability to perform one or more simple computational tasks. Specifically, Avidians may evolve any of nine logic operations, for which they are rewarded with additional computer time to execute and replicate their genomes. Secondarily, speed of replication in Avida is influenced by genome size. Organisms with larger genomes naturally require more computer time and replicate at a slightly slower rate. However, under default settings, this factor is offset by artificially rewarding larger genomes with additional computer time, such that genome size is not under direct selection in most experiments. More detailed descriptions of the software are available elsewhere [33-35].

The evolution of complex features has been a central focus of Avida research, and some of the details are relevant for the present experiments. Whenever an Avidian mutates to perform one of nine computational operations, Avida rewards the lucky organism with a *merit bonus *(increasing its total *merit*). Specifically, this occurs when an organism performs logic operations using strings of bits provided by the Avida software. These operations are analogous to solving simple equations using the input values and then reporting the result. When an organism mutates to perform such an operation, the Avida software multiplies its merit by the corresponding bonus, thereby increasing its replication rate (Table 1). For example, if an organism performs the NAND operation, it will receive a bonus of 2 (fitness effect of 1.0), effectively doubling its relative replication rate (fitness). Organisms are rewarded for each operation only once, i.e., multiple bonuses are not received for performing the same operation multiple times. EQUALS (EQU) is the most complex logic operation rewarded in the Avida environment, conferring a merit bonus of 32 (fitness effect of 31.0).

**Table 1:** Default rewards for performing nine logic operations in Avida

Logic operation	Computation	Number of NAND operations needed (*n*)	Default multiplicative bonus (2^*n*^)	Default fitness effect (*w *- 1)
NOT	~A; ~B	1	2	1.0

NAND	~(A and B)	1	2	1.0

AND	A and B	2	4	3.0

ORNOT	(A or ~B); (~A or B)	2	4	3.0

OR	A or B	3	8	7.0

ANDNOT	(A and ~B); (~A and B)	3	8	7.0

NOR	~A and ~B	4	16	15.0

XOR	(A and ~B) or (~A and B)	4	16	15.0

EQU (XNOR)	(A and B) or (~A and ~B)	5	32	31.0

Default rewards for performing nine logic operations in Avida, adapted from Lenski *et al. *[13]. Complexity (*n*) is measured arbitrarily as the number of NAND operations necessary for performing the logic operation. Combinations of NOT and NAND can be used to construct all other logic operations. Beneficial fitness effects are calculated as *w *- 1, where *w *is the relative fitness of an organism that incurs the mutation of interest.

Avida may be conceptualized as a computational Darwinian search designed to discover the EQU operation. The simplest operations in Avida are easy to evolve, i.e., NAND and NOT are performed by a single genomic instruction, provided instructions for correctly inputting and outputting numbers are present. Any logic operation can be built using different combinations of NAND and NOT. Therefore, EQU can itself be constructed using any of the eight simpler operations as precursors, providing a scalable fitness landscape for the evolution of complexity - beneficial changes are useful for constructing more complex beneficial features. When NAND or NOT arises, the software rewards the lucky organism by doubling its fitness. Fitness bonuses for the other operations increase exponentially with complexity (Table 1). The evolution of EQU may therefore proceed one advantageous step at a time, each step requiring relatively few mutations. Dembski and Marks [36] have suggested the term "stair step active information" to describe this type of reward scheme.

Some of the ways Avida has been implemented (e.g., its parameter settings) are distinctly "un-biological" [33]. These factors include the distribution of mutational fitness effects, the fitness terrain, and the artificial rewards given to organisms with larger genomes. The present study pursues several lines of experimentation with altered mutational fitness effects to improve biological relevance and aid in the interpretation of Avida results. The first set of experiments removed merit bonuses to determine which logic operations arise by mutation alone, without selection. The second set of experiments examined Avida's default settings to quantify typical aspects of evolutionary change in this system. In order to test the hypothesis that mutation pressure prevents the fixation of beneficial operations in Avida, a third set of experiments examined logic operation frequencies at a reduced mutation rate. Finally, a fourth set of experiments implemented fitness effects falling in the normal biological range (0.01 - 1.0), rather than Avida's default range (1.0 - 31.0). The effects on evolutionary dynamics were observed.

## Results

### Mutation and drift

Twenty experiments were performed in which no logic operations were rewarded. Across these experiments, an average of 6.4 (± 0.8) operations drifted into a population at least once over the course of 10,000 generations, indicating that they are easily produced by random mutation. Because of this, a distinction was made between those operations that arose by chance in Avida (those that *arose*) and those that selection was able to propagate (those that *successfully evolved*, i.e., rose to a frequency of 50% or greater, following the precedent of biological studies [37,38]).

Table 2 describes the dynamics of mutational production and drift for specific logic operations (see additional file 1 for further information). Seven of the operations in Avida were produced by random mutation alone, without selection for any beneficial precursors, indicating that they are relatively simple given the instruction set provided in Avida (i.e., Avida's *chemistry *or *physics*). Some of these operations reached appreciable frequencies by drift, and even the relatively complex operation ANDNOT arose in all 20 experiments. The EQU and XOR operations did not arise, indicating that they require advantageous precursors, and are unable to be generated by chance alone given the probabilistic resources of 10,000 generations in Avida, in agreement with results reported elsewhere [13,39]. In light of this, the seven simpler operations are best viewed as alternative potential precursors of XOR and EQU, rather than intermediates in a specific succession of operations.

**Table 2:** Dynamics of mutation and drift for nine logic operations in Avida

Logic operation	Proportion of experiments in which operation arose by mutation	Average maximum frequency in population	Average maximum number of organisms	Maximum frequency observed	Maximum number of organisms observed
NOT	1.0	0.027 (± 0.0062)	97	0.038	134

NAND	1.0	0.017 (± 0.0046)	61	0.028	101

AND	0.95	0.0015 (± 0.00099)	5	0.0036	13

ORNOT	1.0	0.0063 (± 0.0020)	27	0.013	47

OR	0.8	0.00089 (± 0.00091)	3	0.0036	13

ANDNOT	1.0	0.0030 (± 0.0019)	11	0.0072	26

NOR	0.6	0.00053 (± 0.00062)	2	0.0022	8

XOR	0	0 (± 0)	0	0	0

EQU (XNOR)	0	0 (± 0)	0	0	0

Dynamics of mutation and drift for nine logic operations in Avida. Though none of the operations reached high frequencies without a selective advantage, mutation alone produced all operations except XOR and EQU, and many drifted to appreciable frequencies. The simpler operations are best viewed as alternative potential precursors to XOR and EQU.

### Evolution under default settings

**Figure 1:**
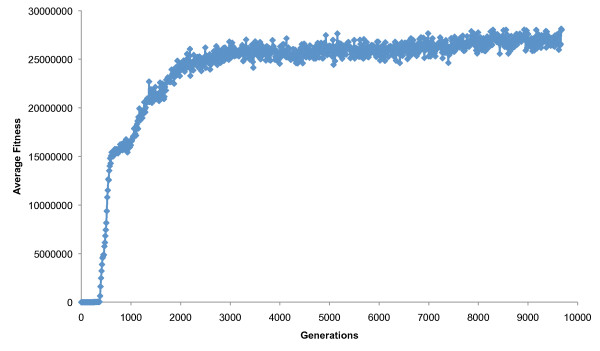
**Trajectory of average fitness in a case study population under default settings**. Fitness reached a maximum as the logic operations approached maximum frequencies. The population reached an end-of-experiment fitness of just under 30 million. Fitness was measured as the merit divided by the generation time, and reported relative to the ancestral organism.

Thirty experiments were performed using Avida's default settings. An average of 8.6 (± 0.7) logic operations successfully evolved. Fitness increased by an average of 19,749,130 (± 14,174,227), corresponding to an average increase of approximately 100.17% per generation, in agreement with results reported elsewhere [13]. The large variance of this estimate results from populations that reached considerably higher fitnesses. Fitness tended to approach a maximum as the logic operations spread through the population (Figure 1), corresponding to the limited availability of high-impact beneficial mutations (i.e., only nine logic operations). See additional file 2 for further information.

### Mutation pressure and clonal interference

Interestingly, no operations reached fixation under default settings, despite their remarkably high fitness bonuses. The average end-of-experiment frequency for operations that successfully evolved was only 84.5% (± 13.5%). This contrasts with the rapid fixation of high-impact beneficial mutations observed in biological experiments. For example, in one study of *E. coli *[37], the *Rbs- *mutation increased fitness only by about 1.4%, yet reached fixation (97-100%) in only 2,000 generations.

We hypothesized that the failure of fixation in Avida is due to mutation pressure resulting from a relatively high mutation rate per genome (0.85). To test this, 30 experiments were performed with a reduced rate of 0.5 mutations per genome per generation to compare end-of-experiment frequencies. Overall logic operation frequencies in the lower mutation environment were significantly (P = 1.84 × 10^-5^) higher, reaching an average frequency of 90.0% (± 14.8%). These differences were individually significant (P < 0.05) for five of the nine operations (Table 3), and all reached higher frequencies in the low mutation environment. Interestingly, an average of only 8.2 (± 0.9) operations evolved in the low-mutation environment, fewer than those in the default environment, but this difference was not highly significant (P = 0.059). Further information is available in additional file 3.

**Table 3:** The effects of mutation rate on phenotype frequencies

Logic operation	Frequency with default mutation rate	Frequency with reduced mutation rate	P-value
NOT	0.93	0.96	0.00018*

NAND	0.91	0.93	0.68

AND	0.77	0.84	0.29

ORNOT	0.87	0.95	0.013*

OR	0.86	0.92	1.7E-07*

ANDNOT	0.88	0.92	0.00017*

NOR	0.85	0.90	8.5E-08*

XOR	0.73	0.77	0.51

EQUALS	0.78	0.83	0.15

The effects of mutation rate on phenotype frequencies. This table shows the average end-of-experiment frequencies for logic operations evolving (1) in the default environment and (2) in an environment with a reduced mutation rate. P-values are for two-tailed two-sample t-tests with equal variances, and significant values are marked with an asterisk*. All calculations used only nonzero frequency values (operations that were not present were not considered).

**Figure 2:**
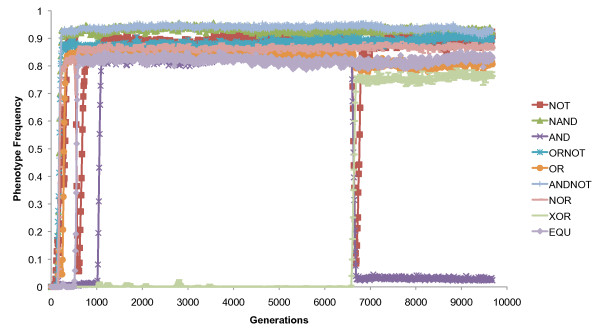
**Phenotype frequencies in a case study population under default settings**. A mutation producing the XOR operation also deactivated NOT and AND around generation 6,580. Clonal interference resulted in the near-extinction of NOT and AND. However, a compensatory mutation restored the NOT operation, and it regained a high frequency.

The competition of different beneficial mutations, known as clonal interference in asexual systems [40], was commonly observed in our study. Because they cannot recombine into a single genotype, such mutations can hinder one another's progress toward fixation, with highly beneficial mutations driving more moderate ones to extinction. For example, in one experiment (Figure 2), a mutation appeared to deactivate the NOT and AND operations (fitness effects of 1.0 and 3.0, respectively) to produce the XOR operation (fitness effect of 15.0) around generation 6,580, driving the former operations to near extinction. The success of XOR followed expectation, because the advantage of XOR exceeds the combined fitness bonuses of NOT and AND. However, because NOT arises very commonly in Avida, a compensatory mutation produced it in the XOR genotype within about 100 generations, allowing it to regain a high frequency in the population.

### Evolutionary consequences of low-impact mutational fitness effects

To explore the evolutionary consequences of low-impact mutational fitness effects in Avida, experiments were performed with multiplicative fitness effects of 0, 0.01, 0.05, 0.075, 0.1, 0.25, 0.5, and 1.0, with 0 being neutral and 1.0 corresponding to a doubling of fitness (100% increase). This allowed an empirical estimation of Avida's selection threshold, the critical "tipping point" between random genetic drift and natural selection. Because most operations arise readily by chance in Avida, evolution of an individual operation was again considered successful only if its end-of-experiment frequency was 50% or greater. Two sets of 20 replicates were performed, one for beneficial mutations and one for deleterious mutations, with each replicate consisting of eight experiments (one experiment for each fitness effect). For beneficial mutations, experiments were simply initiated with uniform fitness effects of the specified value (e.g., for a fitness effect of 0.1, all nine operations multiplied fitness by 1.1). For deleterious mutations, experiments were performed first under Avida's default settings to allow the evolution of complexity, and then continued for an additional 10,000 generations with the alternative beneficial fitness effects. A range of fitness effects could also have been used, with rare operations incurring greater benefits; however, uniform fitness effects were ideal for the purpose of approximating the selection threshold in Avida, and using a range would not appreciably alter our results. Since mutation pressure is a significant force in Avida, it was expected that the existing operations would incur deactivating mutations, and that the fitness bonuses would determine selection's efficacy in maintaining those operations.

**Figure 3:**
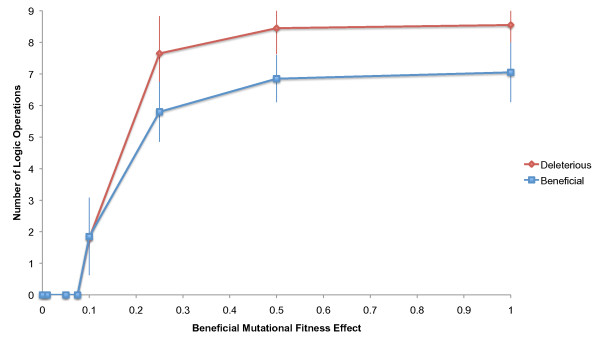
**Selection threshold for mutations affecting fitness**. The number of logic operations evolved or maintained is shown as a function of the beneficial mutational fitness effect used. For beneficial mutations, the end-of-experiment average number of operations was reported; e.g., when logic operations had fitness effects of 0.25, an average of 5.8 operations evolved by positive selection. For deleterious mutations, the number of operations remaining after evolution with alternative fitness effects was used; e.g., when logic operations had beneficial fitness effects of 0.25, an average of 7.65 were maintained by purifying selection. In both cases, the number of operations evolved or maintained was reported relative to the beneficial fitness effect of an operation-creating mutation for simplicity. Deleterious mutations therefore correspond to the reversal of beneficial mutations with the fitness effects indicated on the x-axis. No operations evolved or were maintained for fitness effects of ≤ 0.075. Half of the operations evolved or were maintained at a fitness effect of approximately 0.2.

Results are summarized in Figure 3. Complete selection breakdown occurred for mutational fitness effects in the 0.075 - 0.1 range. No operations were produced or maintained by selection for fitness effects ≤ 0.075, implying that mutations affecting fitness by approximately 7.5% or less are entirely unresponsive to selection in Avida. Both deleterious and beneficial mutations had similar selection thresholds in the range of 0.1 - 0.25, or approximately 0.2, indicating that the fate of mutations affecting fitness by 20% or less in this system is determined primarily by genetic drift, not selection. This threshold is far below the smallest fitness effect implemented in the default settings. Further information is contained in Additional file 4 and Additional file 5.

## Discussion

Although Avida has routinely been used to address biological questions, some aspects of the program are not amenable to direct biological comparison. For example, key terms such as nucleotide, gene, heritability, selection, and fertility lack a clear equivalent in the software. Because of this, several approximations were necessary in this study. Allele frequencies were measured as phenotype frequencies, ignoring the potential for chance performance. Mutation rates were measured as the rate of random substitution of single instructions, though these monomers can perform multiple computations and are not comparable to biological nucleotides. Generation times changed substantially over the course of a typical experiment, so the average end-of-experiment generation time was used to measure experiment length. Finally, genome size also fluctuated in these experiments, causing the genomic mutation rate to change. For simplicity, the mutation rates reported were those for the ancestral genome size (100).

In these experiments, all but two logic operations in Avida arose via mutation alone, despite conferring no fitness rewards (Table 2). Most operations are therefore very simple to produce in the Avida environment, with relatively short waiting times. The genomic monomers (instructions) themselves do most of the computational work that these operations require; this underlying information is included in the artificial physics of Avida and is not subject to mutational change. Interestingly, un-rewarded operations did not accumulate to produce the more complex operations XOR and EQU. This suggests difficulties for traditional models of evolution by gene duplication in which novel functions arise by neofunctionalization of unconstrained loci [41,42]. Previous work has explored the evolution of EQU when other operations are made neutral [13,39], and further Avida studies should explore the dynamics of neutral evolution in digital organisms.

Several studies have focused on the evolution of "robustness" in Avida under elevated mutation rates [25-28,43]. These studies have shown that, when functional genomes experience high mutation rates, functionality is generally lost, with some operations evolving to utilize fewer genomic positions. This is consistent with the results reported here, which suggest that mutation pressure is a significant force preventing the fixation of beneficial genotypes in Avida (Table 3). Reduced mutation rates allowed advantageous phenotypes to reach higher frequencies; however, fewer operations evolved, evidencing a tradeoff between reducing genetic load and increasing the waiting time to beneficial mutation.

The decelerating rate of adaptive change in Avida (Figure 1) is somewhat reminiscent of biological evolution experiments, e.g., with bacteriophage [44] and *E. coli *[45,46]. However, the explosive fitness increases observed in Avida are roughly seven orders of magnitude greater than those observed in biological experiments of similar duration. Because fitness is defined as relative replication rate in Avida, the program's results may be directly compared with those from biological studies. For example, in experiments with *E. coli*, growth rate increased by an average of ~37% after 2,000 generations [47], ~48% after 10,000 generations [45], and ~75% after 20,000 generations [46]. These changes, resulting from numerous mutations, are negligible compared to those observed under Avida's default settings. Yet the fitness leaps observed in Avida are due primarily to the large multiplicative fitness effects of just nine simple innovations. For example, when fitness effects for all logic operations were set to 1.0, the average end-of-experiment fitness plummeted from almost 20,000,000 to just 180 (still an immense increase relative to biological organisms).

An analogy will help to elucidate the preceding point. Consider species A, a large mammal with a generation time of 30 years, and species B, a bacterial species with a generation time of 1 day. In terms of replication rate, species B is about 10,950 times fitter than species A. Yet this number pales in comparison to the increases observed in Avida. After only 10,000 generations, the fitness (replication rate) of digital organisms in Avida increased by 20 million. Such an increase would allow mammalian species A to evolve a generation time of just 1.6 minutes in this time. This phenomenon occurs because the bonuses readily available to digital organisms in Avida are large and multiplicative, producing exorbitant gains in fitness (i.e., the product of all possible bonuses is 2^2 ^× 4^2 ^× 8^2 ^× 16^2 ^× 32 = 33,554,432). Fitness bonuses this large are extremely rare in nature (but see references [48,49]).

Mutations of smaller effect (i.e., fitness effects of ≤ 1.0) can occur in Avida when the generation time is altered by insertions or deletions within an organism's replication loop. However, the rewards gained by performing logic operations dominate fitness dynamics in Avida, and these are the only fitness effects that can be user-specified. Mutations disabling any of the evolved operations have similarly large (but not identical) deleterious effects. It is our view that the distribution of fitness effects used in Avida has severely limited its relevance to biological systems.

Though many details of the biological distribution of mutational fitness effects have yet to be understood [50], a general picture has emerged. There is a continuum of fitness effects and, with few exceptions [51,52], advantageous mutations are exponentially distributed, being much more rare than deleterious mutations [29,30,53-56]. The distribution of deleterious mutations is likely multimodal, with a distinct class being lethal and another class having very small effects [29]. In most systems studied, deleterious mutations of small effect are more abundant than those of large effect [29,54], such that selection coefficients in the range of 0.01 to 0.1 are considered large [48]. For example, over 90% of gene knockouts in *E. coli *are viable [57], decreasing fitness by an average of ≤ 3% [58]. Similarly, a recent study of mutations in *Salmonella typhimurium *[54] reported average deleterious selection coefficients of 0.0096 and 0.0131 for synonymous and nonsynonymous mutations, respectively. No significantly advantageous mutations were found, and no mutations caused a complete loss of function.

Viruses are somewhat peculiar because of their high mutational sensitivity, with approximately 20 to 41% of mutations being lethal, and many mutations being neutral or nearly neutral [30,55,56,59]. However, viable deleterious mutations of small effect are still more common than those of large effect. A recent review [55] of several viruses reported a mean fitness effect of 0.10 to 0.13 (though some estimates have been considerably lower [60]). Lind *et al. *[54] note that the high frequencies of neutral mutations reported in some studies may be a consequence of assays that lack sufficient sensitivity, and that nearly neutral mutations may be more common than previously thought. Some studies have reported the fixation of highly beneficial mutations in viruses [48,49], but they have not consistently measured fitness effects as they are defined here. These reports have suggested that beneficial mutations in viruses may be described by a uniform distribution with an upper bound [49]. It is clear that mutational fitness effects in biological organisms are substantially smaller than those used heretofore in digital life research.

The term *selection threshold *has been introduced to describe the critical mutational fitness effect for which natural selection and random genetic drift contribute equally to a mutation's fate [Gibson P, *et al.*, in preparation]. Though many studies report the occurrence of neutral mutations, it is unlikely that any mutation is truly neutral [29], including those in viruses [30]. It is increasingly being recognized that most mutations in multicellular organisms fall far below the selection threshold, having fitness effects so slight that they cannot be measured [29,49]. It is also noteworthy that highly beneficial mutations have gone undetected in most evolution experiments with eukaryotes [53]. Clearly, the results of evolution experiments with microorganisms cannot be extrapolated to eukaryotes with larger genomes, greater phenotypic complexity, and smaller population sizes. In light of this, some may ask whether the results of experiments with digital organisms have any relevance to living systems. We conclude that digital genetics is a valid platform for studying some biological questions, but that the applicability of results will depend critically upon the parameters used.

Population size has routinely been used as the sole predictor of selection efficacy. To our knowledge, the present study is the first that uses an empirical approach to estimate the selection threshold in an evolving system. This approach implicitly considers all factors affecting selection, including (but not limited to) population size, the probabilistic nature of selection, and environmental effects. We find that, given the sources of noise inherent in the Avida world, mutations with fitness effects below the 0.075 - 0.1 range are entirely invisible to selection, despite arising frequently. Fitness effects of approximately 0.2 are necessary for selection to successfully capture half of the beneficial mutations that arise, corresponding to the selection threshold. Though the value of this threshold is certain to differ among biological and digital systems, its existence has important theoretical and medical implications. Other Avida experiments [39] using single organisms and truncation selection have improved the program's performance, suggesting that local interactions and the probabilistic nature of selection are important sources of noise in the Avida world. Some readers might object that, due to this noise, fitness effects in Avida do not directly correspond to fitness effects in biological organisms. If there is truly no correspondence, then experiments using Avida are not capable of shedding light on biological questions. However, there are several reasons why these experiments are broadly relevant to biology. Importantly, there is also noise in biology. The information contained in the heritable material is processed through multiple levels, including transcription, mRNA processing, protein folding, physiological interactions, and more. Each level is subject to mechanisms of canalization and homeostasis that obscure the effects of mutations on fitness. Further, most noise in Avida may be attributed to the probabilistic nature of selection, yet probability selection is also operative in nature, and may be considerably weaker [61] than the scheme implemented in Avida. It is therefore probable that biological organisms experience more fitness noise than digital organisms.

The present study used uniform mutational fitness effects. A range of fitness effects could also have been used, with rare logic operations incurring greater benefits. However, uniform fitness effects are often employed, and were ideal for the purpose of approximating the selection threshold. Even the simplest operations did not evolve at fitness effects of ≤ 0.075, demonstrating that the threshold exists independent of a mutation's rarity or the length of an evolutionary experiment. Moreover, because each operation is rewarded only once per organism, the evolution of simpler operations should not prevent the subsequent evolution of additional complexity. Further research should attempt to better approximate the selection threshold in this and other systems using varying fitness effect distributions.

The distribution of mutational fitness effects has serious implications for genetic disease. Numerous analyses have confirmed that the accumulation of slightly deleterious mutations can cause gradual fitness loss leading to extinction in asexual species [31,62-66], and similar processes are relevant to sexual species [67,68], including humans [69-74]. The results reported here reveal a quantifiable selection threshold, below which random genetic drift dominates the behavior of advantageous and deleterious mutations alike. Biological studies elucidating the extent of sequence-dependent functionality in nonprotein-coding DNA regions will continue to inform estimates of the rates of mutations affecting fitness in various species, allowing a realistic evaluation of the severity of genetic decline that can be expected in coming generations.

We observed that, when fitness effects in Avida are small, all advantageous logic operations are lost. Though digital organisms are peculiar in that they can survive such a loss, these data confirm that the accumulation of slightly deleterious mutations can lead to decreasing biological functionality and potentially eventual extinction. Because deleterious mutations are much more common than advantageous mutations in most systems studied, reduction in the efficacy of selection imposes strong directionality on evolution by favoring the fixation of deleterious mutations [2]. The conditions under which fitness recovery may be possible [75] should be studied more thoroughly using computational approaches. An understanding of these issues may be applicable to the lethal mutagenesis of pathogen populations [66,76], and is relevant to human health [74]. It is clear that mutation accumulation may affect human health at various levels, including the nuclear and mitochondrial genomes as well as immune cells. For example, mutation accumulation may play a key role in the deterioration of the immune system during HIV infection [77]. It may also influence the longevity of pathogen populations. Various factors relevant to pathogen attenuation, including the consequences of periodic bottlenecking and elevated mutation rates, should be studied using computational models. Understanding the interplay of these co-evolutionary processes may allow for substantial advances in medicine, including novel treatments and an increased awareness of the role of mutation in disease.

## Conclusions

Avida has previously been used as a powerful demonstration of adaptation resulting from high-impact beneficial mutations. However, there are several ways in which Avida's default settings produce results which conflict with observations from biological experiments. Precursors necessary for the most complex logic operation in the program, EQU, are frequently produced by random mutation, yet confer very large fitness rewards. Fitness effects of beneficial mutations under Avida's default settings range from 1.0 to 31.0, values that are extremely rare in the natural world. As a result, fitness increases by an average of 20 million in only 10,000 generations. This is roughly seven orders of magnitude greater than the changes observed in biological evolution experiments.

In contrast to Avida's default settings, most mutations in biological organisms are low-impact [29], and this class of mutations may dominate evolutionary change [1,2]. When Avida is used with more realistic mutational fitness effects, it demonstrates a clear selection threshold. Mutations that influence fitness by approximately 20% or less come to be dominated by random genetic drift. Mutations that affect fitness by 7.5 - 10.0% or less are entirely invisible to selection in this system. These results provide evidence that low-impact mutations can present a substantial barrier to progressive evolution by natural selection. Understanding mutation is of primary importance, as selection depends on the mutational production of new genotypes. Numerous changes that would be beneficial may nevertheless fail to occur because mutation cannot produce them in the time available. Further, it is important for biologists to realistically appraise what selection can and cannot do under various circumstances. Selection may neither be necessary nor sufficient to explain numerous genomic or cellular features of complex organisms [2-4].

Future studies should explore the interaction of low-impact fitness effects with other evolutionary factors, such as alternative fitness terrains [21], to elucidate their synergistic effects on evolutionary dynamics. Additionally, researchers should attempt to further quantify the selection threshold for various systems, and determine the phenotypic consequences of accumulating low-impact mutations. The accumulation of slightly deleterious mutations may pose an important health risk for numerous species, including humans [74], and warrants further study using computational approaches. Reducing the rate of mutation in the human genome may be an important step in fighting genetic disease. Additionally, the connection between mutation accumulation and pathogen attenuation should be studied. Finally, we recommend that future experiments with digital organisms employ more biologically relevant mutational fitness effects.

## Methods

This study used Avida version 2.8.1 [78]. For all experiments, random number seeds were chosen randomly as an integer in the range 1 to 1000000000. These values are reported in the supplemental data. Settings were manipulated using the configuration files present in the Avida home folder.

In Avida, time is measured in arbitrary units called *updates *in which the population is allowed to execute about 30 instructions per organism. The ancestral organism executes 389 instructions per generation to execute and copy its genome. However, generation times change as organisms evolve. Average end-of-experiment generation time under default settings was 310, corresponding to approximately 9,679 generations over 100,000 updates. Thus 10,000 generations was used as an approximation of experiment length, with 1 generation corresponding to roughly 10 updates.

Fitness in Avida is measured as an organism's total merit divided by its generation time. Thus an increase in merit will increase fitness, while an increase in generation time will decrease it. However, this value has no intuitive meaning and the software does not consistently report it, e.g., at generation 0. For consistency and ease of biological comparison, fitness in this study was re-calculated and reported relative to the ancestral organism's fitness. Thus the average fitness of a generation was equivalent to the average merit divided by the product of average generation time and the ancestral organism's fitness (scaling the ancestral organism's fitness to 1.0). Fitness effects of mutations producing the logic operations were measured as *w *- 1, where *w *is the relative fitness of the organism carrying the mutation. Thus a mutation producing the EQU operation, multiplying fitness by 32, had a fitness effect of 31.0.

To study mutation and drift, 20 experiments were performed in which no logic operations were rewarded. Merit bonuses in the environment.cfg file were defined multiplicatively (type = mult) as 1.0 (value = 1.0), corresponding to fitness effects of 0. All other settings maintained their default values. The output file tasks.dat was examined to determine which operations arose in an experiment. As allele frequencies are not reported by Avida, phenotype frequencies were measured as the number of organisms performing a logic operation divided by the total number of organisms, the latter of which is reported in the count.dat file.

Logic operations readily arose independent of selection. In order to distinguish those mutations that selection promoted, a mutation was said to have simply *arisen *until its frequency reached 50%, at which point it was considered to have *successfully evolved*, a measure that has precedent [37,38]. For analysis of end-of-experiment frequencies of logic operations, averages were taken using only nonzero values (operations that did not arise were not considered).

Thirty experiments were performed under default settings. The case study reported in Figure 1 occurred when using a random seed of 574423164. Thirty experiments were also performed with a reduced mutation rate. For these runs, the copy mutation rate was changed from the default of 0.0075 to 0.004 in the avida.cfg file (COPY_MUT_PROB 0.004). Probabilities of insertions and deletions at the time of replication each remained at 0.05. Thus the default mutation rate corresponded to an average rate of 0.85 mutations per genome per generation, and the lower rate to an average of 0.5 mutations per genome per generation. P-values reported in Table 3 were for two-tailed two-sample t-tests with equal variances (homoscedastic) comparing all nonzero end-of-experiment frequencies from the two environments. The case study shown in Figure 2 occurred when using default settings and a random seed of 13903545.

To study the consequences of alternative mutational fitness effects, merit bonuses were modified in the environment.cfg file. Fitness effects of 0, 0.01, 0.05, 0.075, 0.1, 0.25, 0.5, and 1.0 were used, with 0 being neutral and 1.0 corresponding to a doubling of fitness. Uniform effects were used, such that all advantageous operations conferred the same bonus. For example, for a fitness effect of 0.01, merit bonuses were defined multiplicatively (type = mult) as 1.01 (value = 1.01) for all nine operations. Two sets of 20 replicates were performed, one for beneficial mutations and one for deleterious mutations. Each replicate for beneficial mutations consisted of eight experiments, one for each of the fitness effects tested. Each replicate for deleterious mutations consisted of eight similar experiments in which evolution proceeded first under default settings, and then continued an additional 10,000 generations using the alternative fitness effects and a new random number seed. Because fitness bonuses were multiplicative in our experiments, fitness effects would in reality be slightly different for mutations creating and destroying the same operation. For example, a mutation creating an operation with a bonus of 1.25 would have a beneficial fitness effect of (1 - 1.25 / 1.0) = 0.25, but a mutation destroying the same operation would have a deleterious fitness effect of (1 - 1.0 / 1.25) = 0.2. For simplicity, the consequences of deleterious mutations were simply reported (Figure 3) relative to the beneficial fitness effect. Additionally, while deleterious fitness effects could have been indicated as negative values, the absolute values were used for simplicity.

## Competing interests

The authors declare that they have no competing interests.

## Authors' contributions

CWN participated in the design of the study, carried out all experiments, and drafted the manuscript. JCS participated in the design of the study and helped to improve the manuscript. Both authors read and approved the final manuscript.

## Supplementary Material

Additional file 1 **Mutation and drift**. Dynamics of Avidian mutation and drift across 20 experiments in which no logic operations were rewarded. Click here for file

Additional file 2 **Evolution under default settings**. Dynamics of Avidian evolution across 30 experiments using default settings. Click here for file

Additional file 3 **Evolution with reduced mutation rate**. Dynamics of Avidian evolution across 30 experiments in which a genomic mutation rate of 0.5 per generation was used. Click here for file

Additional file 4 **Selection threshold for beneficial mutations**. Dynamics of Avidian evolution across 20 replicates (160 experiments) employing alternative mutational fitness effects of ≤ 1.0. Click here for file

Additional file 5 **Selection threshold for deleterious mutations**. Dynamics of Avidian evolution across 20 replicates (160 experiments) employing alternative beneficial mutational fitness effects of ≤ 1.0. Evolution first occurred under default settings to allow the evolution of logic operations, then continued with alternative mutational fitness effects. Click here for file
